# Human resource management in post-conflict health systems: review of research and knowledge gaps

**DOI:** 10.1186/1752-1505-8-18

**Published:** 2014-10-02

**Authors:** Edward Roome, Joanna Raven, Tim Martineau

**Affiliations:** 1 Department of International Public Health, Liverpool School of Tropical Medicine, Pembroke Place, Liverpool L3 5QA, UK

**Keywords:** Human resource management, Health workforce, Post-conflict, Fragile, Health systems, Reconstruction

## Abstract

In post-conflict settings, severe disruption to health systems invariably leaves populations at high risk of disease and in greater need of health provision than more stable resource-poor countries. The health workforce is often a direct victim of conflict. Effective human resource management (HRM) strategies and policies are critical to addressing the systemic effects of conflict on the health workforce such as flight of human capital, mismatches between skills and service needs, breakdown of pre-service training, and lack of human resource data. This paper reviews published literatures across three functional areas of HRM in post-conflict settings: workforce supply, workforce distribution, and workforce performance. We searched published literatures for articles published in English between 2003 and 2013. The search used context-specific keywords (e.g. post-conflict, reconstruction) in combination with topic-related keywords based on an analytical framework containing the three functional areas of HRM (supply, distribution, and performance) and several corresponding HRM topic areas under these. In addition, the framework includes a number of cross-cutting topics such as leadership and governance, finance, and gender. The literature is growing but still limited. Many publications have focused on health workforce supply issues, including pre-service education and training, pay, and recruitment. Less is known about workforce distribution, especially governance and administrative systems for deployment and incentive policies to redress geographical workforce imbalances. Apart from in-service training, workforce performance is particularly under-researched in the areas of performance-based incentives, management and supervision, work organisation and job design, and performance appraisal. Research is largely on HRM in the early post-conflict period and has relied on secondary data. More primary research is needed across the areas of workforce supply, workforce distribution, and workforce performance. However, this should apply a longer-term focus throughout the different post-conflict phases, while paying attention to key cross-cutting themes such as leadership and governance, gender equity, and task shifting. The research gaps identified should enable future studies to examine how HRM could be used to meet both short and long term objectives for rebuilding health workforces and thereby contribute to achieving more equitable and sustainable health systems outcomes after conflict.

## Introduction

As the international community looks towards achieving universal health coverage (UHC), post-conflict settings are seen as particularly difficult contexts in which to realise better health systems outcomes, such as improved health, equity, social inclusion and trust. In 2013, a record total of 45 violent conflicts occurred globally [1]. Conflict-affected countries often suffer severe disruptions to disease control programmes, interruptions of drugs and medical supplies, destruction of infrastructure, displacement of communities, and flight of health workers [2]. However, long-after conflict ends, populations remain disproportionately at risk of infectious diseases and in even greater need of adequate health provision than non-conflict affected, resource-poor countries [3]. Further, in fragile conflict-affected states (FCAS), health indicators are significantly worse than in non-conflict affected fragile states [4]. Half of all global child deaths occur in fragile states, but only two out of 35 states considered fragile are on track to meet the child mortality target by 2015 [5].

In the growing number of post-conflict countries, where health systems and health workers are often victims of conflict, there is an urgent need to understand how human resource management (HRM) can contribute to health systems rebuilding [2,6,7]. However, the task of rebuilding health systems with competent and equitably distributed health workers is often protracted and fraught with complexities. Post-conflict settings typically transition through three broad and overlapping phases [8]: emergency and stabilisation (the first year post-armed conflict), transition and recovery (1–4 years post-armed conflict), and peace and development (4–10 years post-armed conflict). Nevertheless, reconstruction is rarely a linear process and about 40 per cent of countries relapse into conflict [9]. Health systems reconstruction is highly dependent on the ability of governments to first re-establish security and reconciliation; rebuild trust in state institutions; promote unity; and reinstate the rule of law.

Some of the most important decisions to steer the long-term path of health systems rebuilding are made in the early post-conflict “moment”. Decisions related to the management and development of the health workforce are critical, but present a particular challenge to new or transitional governments – especially those of fragile states with weak capacity and legitimacy. HRM policies and strategies must effectively address the many systemic effects of conflict on the supply, distribution and performance of the health workforce. In addition to flight of human capital, these include: mismatches between skills and service needs; salary distortions engendered by the influx of non-state employers; breakdown of pre-service training; lack of human resources (HR) data to inform workforce planning; lack of management capacity at different health systems levels; and underperformance of the remaining health workforce. Of course, not all these problems result directly from conflict; some are residual from pre-conflict times and exacerbated by conflict [10].

This paper presents a global review of published research on HRM in post-conflict health systems in the past decade (2003–2013). Despite the growing interest and body of research in this area, there have been few reviews that synthesise the available evidence. An exception is Fujita et al. [11] who identified lessons from three post-conflict states to build a model of human resources for health (HRH) systems development. The present review differs in two ways. First, it synthesises topics and findings in published studies and reports across a wide range of post-conflict settings, using an analytical HRM framework to guide and structure the review. Second, it uses this framework and the findings to identify important research gaps that will enable future studies to build the evidence base on HRM in post-conflict health systems.

## Methods

HRM is a complex area of management and its role in health service delivery has grown more difficult [6]. To guide and structure this literature review, we used an analytical framework that summarises important topics in HRM (Table 1). The framework, adapted from Martinez and Martineau [12], outlines three functional areas of HRM – workforce supply, workforce distribution, and workforce performance – and their corresponding topic areas. The framework includes a number of cross-cutting topics that relate to more than one of these functional or topic areas: for example, the rationale of task shifting is to mitigate worker shortages (workforce supply) by reorganising tasks among the remaining workforce to improve service delivery (workforce performance). The cross-cutting topics were based on Dal Poz et al. [13] with the addition of gender [14].

**Table 1:** Framework for analysing HRM publications

	**Workforce supply**	**Workforce distribution**	**Workforce performance**
Topic areas	● Recruitment and selection	● Deployment (including incentives)	● Work organisation and job design
	● Pay		● Management and supervision
	● Pre-service education and training		● Performance appraisal
			● Performance-related incentives
			● In-service training
Cross-cutting topics	● Retention		
	● Task shifting		
	● HR data		
	● Leadership and governance		
	● Finance		
	● NGOs and aid agencies		
	● Gender		

We searched PubMed, Google Scholar, Science Direct and the Cochrane database for articles published in English in the past decade (2003–2013). Institutional reports were sought from websites including the World Health Organization, the World Bank, Health and Fragile States Network, and the United States Institute of Peace. The search protocol used three context-specific keywords – post-conflict, reconstruction, and fragile – in combination with the following topic-related keywords: human resources, management, workers, workforce, recruitment, attraction, retention, employment, deployment, posting, task shifting, training, development, skills, performance, pay, salaries, supervision, incentives, motivation, migration, leadership, information systems, expatriate, donors, NGO, and gender.

Publications were searched in early 2014 and were required to meet two inclusion criteria: (1) At least one of the context-specific keywords (or synonym of) is covered and (2) at least one of the topic-related keywords (or synonym of) features as an important focus or discussion point. We first screened publication titles and abstracts and removed any publications that did not meet the criteria. We then retrieved full texts for the remaining publications and screened again using the topic-related keywords. Within-publication references were checked to identify additional sources. The review was desk-based and therefore ethical approval was not sought.

## Results

A total of 56 publications met the inclusion criteria. The functional areas of workforce supply and workforce performance were almost equally represented, whereas workforce distribution (deployment) was covered by less than a third of publications (see Table 2). The most popular topic areas relate to training – both pre-service to increase supply and in-service to improve performance. The next three most popular topic areas relate to workforce supply and distribution, with the remainder being related to workforce performance.

**Table 2:** Frequency of HRM topic areas and coverage of HRM functional areas (publications n = 56)

**HRM topic areas**	**No. of publications**	**%**	**WS**	**WD**	**WP**
Pre-service education and training	24	42.9	24		
In-service training	22	39.3			22
Pay	19	33.9	19		
Recruitment and selection	16	28.6	16		
Deployment	16	28.6		16	
Performance-related incentives	11	19.6			11
Management and supervision	11	19.6			11
Work organisation and job design	10	17.9			10
Performance appraisal	2	3.6			2
		Totals	59	16	56

Key for HRM functional areas: WS = workforce supply; WD = workforce distribution; WP = workforce performance.

The majority of publications (63%) were based on secondary data (see Table 3) – of these, case study analysis was the most common study type. The small number of primary data studies were based on an almost even number of quantitative, qualitative and mixed methods.

**Table 3:** Publications by type of data/study (publications n = 56)

**Type of data/study**		**No. of publications**	**%**	**No. of publications**	**%**
Primary data		13	23.2		
Quantitative				4	7.1
Qualitative				5	8.9
Mixed				4	7.1
Secondary data		35	62.5		
Literature review or commentary				12	21.4
Institutional report, guidelines or policy brief				6	10.7
Case study analysis				17	30.4
Mixed primary and secondary data		8	14.3	(8)	(14.3)
Totals		56	100	56	100

The remainder of this section reports on findings against the three functional areas of HRM in Table 1, the corresponding HRM topic areas, and the cross-cutting topics where found.

### Workforce supply

#### Recruitment and selection

Studies on recruitment and selection have focused mainly on the difficulties of attracting trained or qualified health workers during the early post-conflict period. First, it is common for national health systems to become targets during conflict [2], resulting in attacks against health workers and consequently flight of human capital (e.g. Cambodia, Mozambique, Rwanda) [11,15]. In some instances, flight of health workers may continue post-conflict on political grounds (e.g. East Timor and Kosovo) [16]. In addition to frontline workers, numbers of health support staff may also reduce, further impeding efforts to resume service delivery [2].

Second, the breakdown of health services during conflict typically creates mismatches between skills and service needs. Pavignani [17] argued that military health services grow during conflict and compete directly with the public health sector to attract trained health workers. Warring factions may use forcibly recruited or politically affiliated workers to run their own health services [17]. When conflict ends, the public sector may absorb these health workers to meet immediate service needs. However, this is often done with little regard for the appropriateness of workers’ skills and the quality of training received during conflict [17]. Expatriates are often brought in by NGOs and aid agencies to fill gaps in local health workforces. Although often more willing than local staff to work in post-conflict areas [18], common problems in expatriate recruitment include high turnover; variable skills and qualifications; resentment over control and salary differentials; and failure to transfer skills to national staff [17,18].

Third, WHO [19] asserts that effective leadership is critical to rebuilding workforce supplies post-conflict. Good leadership helps ensure the commitment of major actors in reaching a consensus on human resource planning criteria and standards, salary scales, and contracts. However, weak governance and political interference in recruitment of health cadres frequently undermines early reconstruction efforts. In Afghanistan, the Priority Reform and Restructuring (PRR) process setup in 2004 was intended to facilitate recruitment of competent staff across the government using updated job descriptions and revised salary scales. However, political interference such as nepotism and patronage became a barrier to success [11]. Similarly, when the war ended in Rwanda, ministries were allocated to political parties, but party members filled lower level positions with unqualified and inexperienced staff. The situation improved marginally when the Public Service Commission was established in 2002 to enhance objectivity and integrity in recruitment [20].

Last, the availability of HR data is vital to informing recruitment and workforce planning decisions in the post-conflict period. However, as conflict often results in damage to ministries and health facility infrastructure, such data are often lacking [2]. Fortunately, in the case of Timor-Leste, manual health personnel records were rescued from the Department of Health as it was being burned. These were later transferred to a computerised database to help verify qualifications for recruitment and workforce planning [6]. Health authorities in Palestine implemented a comprehensive computerised personnel information system with information on staff types, numbers, distributions, and qualifications, which supported recruitment and planning processes [21]. However, Smith and Kolehmainen-Aitken [22] argued that enthusiasm for such systems must be matched with a clear focus on how to use and disseminate this information to improve recruitment and workforce planning post-conflict.

#### Pay

The effects of pay on supplies of health workers can be profound and different institutional actors may induce unintended consequences in the labour market. NGOs and aid agencies, for example, can easily attract public health workers using lucrative employment packages [23]. Competition between agencies further distorts pay differentials [24] and accelerates brain drain from the public sector [23]. This causes significant shifts in labour market dynamics and reduces the number of health workers available to rebuild routine public health services – which arguably should be the main goal of post-conflict reconstruction. Many lowly paid public health workers not contracted by NGOs and aid agencies moonlight in private practice, teaching or research to supplement their livelihoods [20]. Levels of service provided to the primary public employer invariably suffer as a result of low productivity and absenteeism [23]. Weak governance and poor regulation in the early post-conflict period often creates space for private health providers to proliferate, uncontrollably in some cases (e.g. Angola) [24]. In Mozambique, the Ministry of Health’s *Health Manpower Development Plan (1992–2002)* had failed to recognise that the pre-war dominance of the public sector would be challenged by a growing market for private health care [24]. In Somaliland, the government’s inability to pay regular and competitive salaries has constrained capacity building in public health facilities and risks jeopardising programmes to train and support health workers [25].

Pay reforms are commonly enacted during reconstruction as a strategy to attract and retain public health workers. Nonetheless, there are several factors that impede their success. In Liberia in 2009, the Ministry of Health sought to increase the number of trained health workers by standardising pay and raising monthly salaries for nurses from 900 Liberian Dollars (US$ 13) to 7590 Liberian Dollars (US$ 108). However, a lack of resources and a public sector wide employment ban undermined the intended benefits [26]. More generally, it is argued that post-conflict pay reforms tend to be ineffective for two reasons: first, those responsible for implementing pay reforms are themselves learning on the job, and second, salary scales are constrained by the amount of donor financing available [22]. In Cambodia, despite an annual average increase in public sector salaries of 20 per cent since 2003, health workers and their families have struggled to live due to the country’s high cost of living [11].

Another impetus for pay reform is to rid the public payroll of ghost workers in order to make better use of existing salary budgets. Incomplete, flawed or manipulated HR data enable some individuals not working in the system to benefit from a salary [26]. In Mozambique the Ministry of Health had removed some 2,000 ghost workers from the payroll up to 2003 [24]. In post-conflict Sierra Leone, the civil service identified 750 ghost workers and more than 1,000 workers over the statutory retirement age [27].

#### Pre-service education and training

Pre-service education and training was a recurrent topic that emerged from the literature. The effect of conflict on the number and types of health workers can be unpredictable: it may cause under-supply (e.g. Cambodia, East Timor, Mozambique) or over-supply (e.g. Angola, Democratic Republic of Congo, Sudan) of different cadres, as well as inconsistencies in levels of competence and expertise within cadres [15]. In situations of over-supply, an immediate focus on improving the quality of the existing health workforce through in-service re-training may be most appropriate [17]. In cases of under-supply, a focus on rebuilding and upgrading pre-service education and training provision is often seen as a viable solution to scaling up health worker numbers. However, there are several challenges to achieving scale-up objectives. First, HRH plans and scale-up strategies are often informed by scant HR data about the status and composition of the current workforce [28]. In Afghanistan, the first HRH development plans drafted in 2003 turned out to be rather vague, as policy makers had no concrete HR data on how many health workers required testing and certification, what their professional categories were, or where they were based [22]. Without such data, it is difficult to gauge the extent to which pre-service training and scale-up strategies will meet demand for health services.

Second, training institutions invariably lose teachers during conflict, some of whom are replaced post-conflict with less qualified staff [29]. Governments often severely underestimate the effects of conflict or crisis on teaching capacity. In post-crisis Zimbabwe, for example, the government pursued an ambitious scale-up strategy that encouraged training facilities to double enrolment, even though 60 per cent of academic and training positions at medical and nursing schools remained vacant [30]. In conflict-affected areas, it is common for NGOs and local providers to fill some of the pre-service training gaps using emergency on-the-job training. However, these training services have been criticised for being unsuited to specific contexts, inefficiently absorbing resources, and having negligible impact [16]. Moreover, reliance by governments on non-public providers can distract from efforts to build long-term education and training strategies [15].

Third, a lack of national standards and quality assurance in education and training institutions has serious implications for the quality of health workers produced. With few qualified teachers working with few or no training resources, learning may be based on what teachers know rather than what students should know [6]. In South Sudan, none of the 36 training facilities offer structured postgraduate training and a unified accreditation system does not exist. About one in ten graduates in the country are deemed unsafe practitioners [30]. Furthermore, in many post-conflict settings, training standards and quality assurance are compromised by unregulated privatisation of training providers [31]. Many of the students who graduate from these private providers expect to be absorbed by the public sector, even when supply far outstrips demand [17].

### Workforce distribution

#### Deployment

Formal deployment systems are critical to achieving equitable health provision. These systems should govern decisions about how to assign health workers to jobs (deployment/posting) and how to transfer staff between jobs and locations (redeployment/secondment). Deployment policies and strategies should in principle be based on analyses of service needs, and current and projected supplies of health workers – although weak governance and patronage can influence transfer decisions [32]. Responsibility for deployment depends on the levels of authority of those managing the systems, which may be coordinated centrally or overseen by district health managers in decentralised environments.

During conflict, deployment systems become weaker or may breakdown entirely. These disruptions engender serious bottlenecks to achieving rapid scale-up and equitable distribution of health workforces. Policies used in more stable settings may be rendered less effective in post-conflict environments – a problem that is not easily remedied [16]. For example, in stable settings, training is strategically linked to deployment of newly trained workers. However, in post-conflict settings, ineffective HR planning and monitoring can cause deployment and training to become “unlinked”, leading to uncontrolled production, as happened in the Democratic Republic of Congo (DRC) [11].

A pervasive problem is the concentration of health workers in areas that are perceived to be safer or have better prospects, which can leave remote and conflict-affected zones underserved. Nonetheless, redeployment of health workers to these areas may be directly hampered by war-related destruction of health facilities and staff houses [16]. NGOs and aid agencies may also contribute to staff being concentrated in more secure areas. Due to operational convenience and security concerns, NGOs in Afghanistan and South Sudan tended to recruit local staff to work in facilities near secure borders [16].

In some societies, cultural norms and gender roles may exacerbate maldistribution of health workers after conflict. In Afghanistan, despite the existence of a formal deployment system for graduates of the community midwifery education programme, non-acceptance of pre-assigned jobs is common – even among committed students [29]. Midwifery students are often warned not to accept pre-assigned jobs in areas of unfolding instability because they are female and at risk of being killed [29].

In addition to direct financial incentives such as pay and bonuses, various other types of indirect financial and non-financial incentives have been used to attract health workers to rural and less secure areas after training, and to promote redeployment of workers from overstaffed to understaffed facilities. Indirect financial incentives include scholarships, free or subsidised housing and schooling, and other allowances [21,26]. Non-financial incentives include career development opportunities; good working conditions; and involvement in decision-making [26,33]. Huicho et al. [34] evaluated job preferences of public sector nurses and midwives in Ayacucho, Peru (an area still recovering from armed conflict in the 1980s and 1990s). The most attractive package to uptake rural jobs was a 75% increase in salary plus a scholarship for a specialisation. Policy simulations showed that this combination could increase rural job acceptance from 36 to 60 per cent.

In the early post-conflict period, when health systems are particularly fragile, MacKinnon and MacLaren [30] argued that short-term, non-financial incentives to work in rural areas can be identified as entry points for national governments, development partners and donors. However, the practice of using incentives to aid deployment depends on the availability of reasonably accurate HR data to identify understaffed areas and movements of individual staff in post.

### Workforce performance

#### Work organisation and job design

Throughout the early post-conflict period, work may need to be reorganised and jobs redesigned to ensure that health workers perform adequately and meet changing service needs [19]. Various health systems actors are involved in facilitating these processes including policy makers, NGOs and aid agencies and health facility managers. Two related topics among the few studies found in this area were job descriptions and task shifting. First, job descriptions (an important HRM tool to define and standardise roles and to assess performance) may have become irrelevant during conflict. When conflict ends, NGOs and aid agencies working in health facilities may draft new job descriptions to meet immediate service needs. However, if these are not centrally coordinated, performance management becomes difficult and a large number of different job descriptions proliferate [6]. In Liberia, although job descriptions were eventually standardised across all cadres, they were ineffectively communicated to staff, leaving workers to pick up tasks informally [26].

Second, task shifting is used to mitigate shortages of trained and qualified health workers by redistributing tasks from trained health workers to those with less training and fewer (or no) qualifications [35], including community health workers [36]. However, without clear policies and job descriptions nor sufficient supplies and equipment, task shifting may put service quality and safety at risk [26]. In Afghanistan, for example, increased demand for midwifery services resulted in unmanageable workloads and health workers being asked to perform unpaid tasks beyond their training [29,37]. Thus, although task shifting seemingly provides a short-term solution to supply shortages and work reorganisation, evidence is still wanting on its effects on overburdened health workers’ productivity levels and competence to deliver services safely.

#### Management and supervision

Strong management capacity at all levels of the health system, including frontline supervision, is vital to improving work performance in areas recovering from conflict [6]. The types of management and supervision activities identified as being important include on-going supportive supervision [3,38], coaching and mentorship programmes [39], in-service training [31], and performance appraisal [21]. During and immediately after conflict, numbers of managers and supervisors capable of implementing these activities reach critically low levels, and are usually non-existent in rural areas [24]. However, these issues are rarely prioritised in long-term training and capacity building strategies [40]. Consequently, untrained, under-resourced and often unsupported managers – who themselves may be coping with the effects of conflict – are tasked with redressing perpetual workforce problems such as low productivity, incompetence, and absenteeism.

Several studies have examined policies and interventions to strengthen management and supervision capacity in post-conflict settings. Health management strengthening was one of the first areas addressed by donors and the United Nations Administrative Mission in Kosovo (UNMIK). UNMIK supported primary health care by sending potential leaders abroad for short-term management training, and provided several hospitals with international managers. Meanwhile, donors organised short courses for up to 300 managers. Although management of health care facilities in Kosovo has come a long way, and health workers now have modern job descriptions, the health care system still suffers from inadequate human resource management [41].

In Liberia, Yale University partnered with local universities to deliver a six-month programme in health management skills [40]. Health managers self-reported substantial improvements in management skills including strategic problem solving, financial management, human resource management, and leadership. Recommended best practices for implementing participatory management strengthening programmes in post-conflict settings included use of short-course formats with practical tools; use of didactic training, on-site projects, and mentoring; and securing ministry-level support to ensure participation [40]. Nonetheless, opportunities to up-skill management during post-conflict reconstruction are sometimes overlooked. In Mozambique, the Ministry of Health’s training plan had strongly recommended training of professional health administrators. This was ignored, however, leaving medical doctors without management skills to run the National Health Service [42].

Finally, on-going research by the ReBUILD consortium in Northern Uganda has raised questions about how managers can better support and protect health workers who witnessed or suffered direct trauma during conflict [43]. However, published research is somewhat silent on the issue of gender-sensitive HRM policies in traumatised areas, where staff are often predominantly mid-level and female. Research on policies to promote other aspects of equal opportunities (e.g. policies sensitive to ethnic minorities) is similarly absent from the literature.

#### Performance appraisal

Performance appraisal is the process through which supervisors review workers’ performance against set targets and responsibilities outlined in jobs descriptions. It is normally used to provide feedback, issue rewards or sanctions, or identify training and development needs. Despite being an important area of performance management, only two publications mentioned staff appraisal. Hamdan and Defever’s [21] analysis of HRH in Palestine between 1994 and 2001 noted how inappropriate performance appraisal systems had negatively affected the efficiency and effectiveness of public health services – although modified performance appraisal systems had been piloted in some health districts. WHO [6] suggests that regular appraisal should complement supportive supervision and training to ensure workers perform at the required levels based on job descriptions.

#### Performance-related incentives

As with attraction and deployment related incentives, performance-related incentives can be financial and non-financial. The function of performance-related incentives is to influence health workers’ levels of satisfaction, motivation and commitment, and therefore their performance in the job. Some incentives may promote intrinsic motivation to perform such as regular praise and recognition, opportunities to develop and use new skills, and participation in decision making. However, as Chee et al. [44] argued, while it is easy to train more workers, it is more challenging to design and implement policies related to employment conditions and incentives that target poor performance.

Although several studies mentioned performance-related incentives, only a few had performance-related incentives as a main focus. El-Jardali et al. [33] investigated the relationship between intrinsic rewards and retention of nurses in Lebanon. The authors observed that opportunities to influence decisions about the work environment was an important factor in nurses’ intentions to stay in the job. A follow-up study found that nurses were particularly satisfied with co-worker relationships and development opportunities, however they were dissatisfied with work scheduling and work-life balance [45]. Further, a study of community health workers (CHWs) by Glenton et al. [46] highlighted several sources of motivation and empowerment to perform well including community recognition, indirect financial incentives such as free health care and education, and involvement in local health facility operations and management committees. Scarcely any studies have discussed direct financial incentives in-depth from a performance perspective in post-conflict settings.

#### In-service training

Ad hoc training provided by NGOs, aid agencies and local providers is often criticised as being of poor quality and having negligible impact [16,17]. As such, the majority of workers surviving conflict need intensive and sustained retraining and up-skilling – a fact that decision makers frequently overlook [24]. In South Sudan, in-service training per staff has been estimated at less than one day for every 10 years of service [30]. NGOs and aid agencies commonly provide essential in-service training and bedside teaching. This is usually conducted informally without coordination with ministries, and therefore lacks certification and accreditation [47]. In the DRC, very few public sector health workers receive in-service medical training unless funded specifically by donors. Consequently, they have almost no exposure to up-to-date medical knowledge [48]. In contrast, the Liberian government has worked closely with donors and health partners to develop pre- and in-service training programmes and institutions between 2006 and 2009, which were carefully aligned to meet long-term objectives [30].

Several studies were found specifically on in-service training programmes and their effectiveness in post-conflict settings. O’Hanlon and Budosan [49] reported a six-month International Medical Corps-led retraining programme for physicians in Kosovo in 1999 and 2000. Despite not having access to foreign medical literatures for almost a decade, participants scored well on an end-of-course knowledge test. In Afghanistan in mid-2001, HealthNet International implemented a high-quality community based midwife training programme. The course prompted national debate about the quality of midwifery education in the country, which led to the introduction of an updated midwifery curriculum in 2003. However, uptake of the new curriculum varied considerably across the country [47].

In Somaliland, an in-service training programme (Life Saving Skills in Emergency Obstetric and Newborn Care) led to a 50 per cent increase in knowledge and 100 per cent improvement in skills assessed. Lack of supplies, medical equipment, and supportive policies were identified as potential barriers to new skills use in this otherwise successful programme [50]. Angola began experimenting with its in-service training system in 1998, but an evaluation of the system in 2005 found that it had failed to provide an adequate return on investment because of poor working conditions and inadequate management practices [24].

## Discussion

To guide and structure this review of human resources for health in post-conflict settings, we used an analytical framework that focused on three functional areas of human resource management (HRM): workforce supply, workforce distribution, and workforce performance. Due to the potential for overlap when categorising HRM topics, we acknowledge the limitations of trying to create a ‘definitive’ framework to organise and analyse topics. Publications between 2003 and 2013 were sought that explicitly considered the post-conflict context. As the extent to which publications considered this varied substantially, the authors inevitably exercised some degree of judgement in publication selection. Furthermore, due to the potential for some countries and regions to relapse into violent conflict, we recognise the limitations of the term ‘post-conflict’. In this section, we discuss the findings in relation to our analytical framework. Table 4 presents a non-exhaustive summary of suggested areas for future research [cf. 2]. These research areas were selected for their specific relevance to HRM and health systems development in post-conflict settings (rather than resource-poor settings in general), and to fill key gaps shown by a comparison of the current knowledge against our analytical framework.

**Table 4:** Suggested areas for future research

**HRM functional area/topic area**	**Areas for future research**	**Rationale**
*Workforce supply*		
Recruitment and selection	● Strategies to assess health workers’ knowledge and skills to facilitate their reintegration into the public health workforce	● Appropriateness of skills of reintegrated health workers is often overlooked
	● Implementation of ‘basic’ HR data systems at an early stage, which can be further developed	● Important to support workforce distribution and performance
	● Equal opportunities including gender-equitable and ethnically sensitive policies to recruit and support health workers in conflict-affected areas	● Evidence on gender-equitable and ethnically sensitive policies is lacking
Pay	● How to implement pay reforms effectively under new post-conflict leadership and governance while minimising unintended consequences for the health workforce and wider health system	● Post-conflict pay reforms risk failing to meet their intended objectives of attracting, motivating and retaining health workers
Pre-service education and training	● Sustainable strategies and policies to attract, train and support qualified trainers and educators after conflict	● Lack of qualified trainers and educators undermines rapid scale-up strategies
*Workforce distribution*		
Deployment	● Opportunities for strengthening governance and administration of deployment in the crucial post-conflict moment and ensuring linkages with training	● Weak governance creates scope for interference in deployment; lack of evidence on administrative systems for deployment; deployment and training systems become unlinked during conflict
	● Financial and non-financial incentives to attract and retain health workers in rural and conflict-affected areas within a competitive incentive environment	● Large influx of non-state employers post-conflict offering attractive salaries and increasing the competition for skilled health workers; conflict-affected rural areas particularly unattractive
*Workforce performance*		
Work organisation and job design	● Approaches to reviewing overall workloads and reallocating work to different cadres to address near-term shortages, but which support longer-term planning	● Few published studies addressing work reorganisation and job redesign at different stages post-conflict
	● Unintended consequences of task shifting on health workers, service provision and utilisation, and the wider health system	● Longer-term effects of formal and informal task shifting are unknown
	● Use of coordinated stakeholder approach to develop interim job descriptions	● Job descriptions may have become irrelevant during conflict; NGO-introduced job descriptions proliferate after conflict and are often uncoordinated
Management and supervision	● Interventions to support health workers affected by conflict to perform well and contribute to safe and effective service delivery	● Health workers targeted during violent conflict may need psychosocial support, but managers may be untrained and themselves affected by conflict
Performance appraisal	Development of basic performance appraisal systems that could be advanced as HRM systems become more formalised and governance strengthened	● Very limited evidence on performance appraisal in post-conflict settings
Performance-related incentives	● Understanding the impact of financial and non-financial incentives on different facets of performance (e.g. productivity, competence, availability) in changing employment contexts	● Incentives used by NGOs in the immediate post-conflict period may impact on the ability of public sector employers to use comparable incentives in the longer term
In-service training	● Understanding how wider health system factors can facilitate or constrain efforts to scale-up in-service training interventions after conflict	● Inadequate funding, lack of supplies and equipment, poor working conditions etc. hinder effective provision of new or upgraded skills

Despite increasing recognition of the need to understand and strengthen health systems reconstruction in post-conflict settings, relatively few studies overall were found on HRM in these contexts. Based on the analytical framework, the most prevalent topic to emerge relates to training – both pre-service and in-service. This is perhaps understandable given the perceived importance of training to achieving workforce scale-up and to resuming service delivery. A focus on short-term, immediate solutions, such as training, to address workforce shortages seems to be common to many post-conflict HR plans. Moreover, in resource-poor post-conflict settings, training provision may have been substandard prior to conflict, and therefore national decision makers in conjunction with international partners may seize opportunities in the early post-conflict period to redress these deficiencies. Nonetheless, many of the prerequisites for ensuring that training supports ambitious scale-up strategies tend to be overlooked, in particular teaching capacity and quality assurance. In the long term, this may require costly retraining to ensure the quality and performance of the workforce.

Pay, a factor in workforce supply, has received some coverage in the literature. Studies have discussed the proliferation of non-state employers in the labour market and the effects on salaries and competition for public sector workers. For example, the public sector frequently depends on non-state actors to attract and retain health workers (e.g. salaries linked to donor financing), while non-state employers such as NGOs use high salaries to attract public health workers to serve priority areas. Topics related to recruitment have also received moderate attention including challenges of reintegrating workers back into the public sector and lack of foresight in aligning skills with service needs. In post-conflict settings, there is an immediate need to recruit competent and skilled staff. Lessons from Rwanda reinforce the imperative to establish objective, merit-based recruitment policies quickly to minimise the consequences of political interference, nepotism, patronage and tribalism [20].

Few publications discussed deployment as a focal issue in post-conflict settings. Some studies point to the problem of maldistribution of different cadres of health workers between urban and rural regions, and between more and less secure areas. Others highlight the types of financial and non-financial incentives required to address these imbalances – although it is difficult to draw clear conclusions on the effectiveness of these deployment incentives due to the wide variety of contextual factors involved. However, there is a notable lack of research on administrative systems for deployment in post-conflict settings, including how and when systems are implemented, and potential consequences of non-enforcement and manipulation of deployment policies for rebuilding health systems.

Discounting in-service training, workforce performance is a relatively neglected area. Topic areas including performance-related incentives (especially financial related); management and supervision; and the reorganisation of work and redesign of jobs have all received little attention. In practice, the perceived urgency among decision makers to achieve workforce scale-up may have overshadowed the ‘latent’ objective of ensuring that health workers are well-managed and perform adequately.

In terms of the cross-cutting topics, lack of reliable HR data is well-recognised as a major hurdle in assessing the state of the health workforce after conflict [28], which in turn hinders implementation of appropriate policies and strategies to rebuild it. The role of NGOs and aid agencies as influential actors in health workforce reconstruction is widely observed – both their roles as enablers (e.g. providing immediate care and emergency training) and constrainers (e.g. attracting away public sector staff, contributing to widening salary differentials, providing in-service training of variable quality). Similarly, the need to strengthen leadership and governance has been highlighted in terms of ensuring transparency and fairness in HR policies and systems, as well as research on interventions to build management capacity at various levels of the health system. Developing strong governance is critical to ensuring the long-term effectiveness and sustainability of all HRM and HRD components after conflict [51,52], and thus more research on HRH governance in both centralised and decentralised settings is warranted. Although lack of a fully legitimate government often constrains the development of accountable health systems, it may be possible, however, to develop HRH policies in states with weak governance provided there is a political environment that is willing to compromise [53]. When no legitimate government exists, the function of stewardship may be pooled among multiple stakeholders, although effective coordination is essential [54].

There is little research on gender equity in HRM, in particular policies to support women health workers who may have been more adversely affected by violent conflict than men. Gender issues tend to be restricted to studies on nurses and midwives, while the wider implications of gender equity after conflict for the health workforce have been overlooked. There is also little research on other aspects of equal opportunities such as those related to ethnicity. Similarly, very few publications have examined task shifting in-depth through a post-conflict lens – for example, how work is reorganised after conflict and the short- and long-term effects of formal and informal task shifting on health worker performance.

The temporal dimension of post-conflict for understanding and explaining how HRM could contribute to sustainable health systems reconstruction has not been thoroughly considered (although one very recent study published in 2014 has examined the phases of HR policy development from the end of conflict [55]). Publications have tended to focus on HRM in the immediate post-conflict period. Witter [56] found a similar situation in her review of research on health financing in post-conflict and fragile states. To understand how decisions made in the post-conflict moment shape the long-term trajectory of rebuilding health workforces, studies should use longitudinal research designs to examine changes in, and determinants of, HRM throughout each phase of reconstruction: from emergency and stabilisation, followed by transition and recovery, and into peace and development [8]. Further, a significant number of publications are based on secondary data, drawing on country case studies, reviews, authors’ observations from working in the field, and prescriptive institutional reports. Although insightful, this perhaps reflects the challenges of conducting high-quality, first-hand research in post-conflict settings, as Witter [56] also noted. When more first-hand research using longer term perspectives can be carried out, it will be possible to analyse HR policy and practice in more depth to determine the conditions for successfully rebuilding health systems and health workforces in post-conflict settings.

## Conclusions

There is a growing but still limited evidence base on human resource management in post-conflict health systems. Much of what we know relates to health workforce supply issues, especially pre-service education and training, pay, and recruitment and selection – although the field could still benefit from further analysis and comparison in these areas. Moreover, future reviews might want to consider findings from grey literatures as well as published studies. This review highlights the need for more in-depth, first-hand research on the other two functional areas of HRM: workforce distribution (deployment) and workforce performance (especially incentives, management and supervision, work organisation and job design, and performance appraisal – and to a lesser extent, in-service training). Future studies should examine these areas across the different post-conflict phases to understand how early HRM decisions shape longer term health systems outcomes. Particular attention should be paid to key cross-cutting topics including gender equity, task shifting to optimise service delivery, and leadership and governance. Without a strong focus on HRH governance at central, provincial and district levels, individual components of workforce supply, distribution, and performance cannot be coordinated and managed effectively post-conflict. Enhanced knowledge across all these areas could inform the strategies that national and international policy makers adopt to achieving universal health coverage in conflict-affected settings, while helping to avoid unintended consequences along the way. It may also distill useful lessons on HRM and health systems strengthening in more developed, stable countries.

## Competing interests

The authors declare that they have no competing interests.

## Authors’ contributions

All authors designed the review. ER carried out the literature search. ER and TM wrote the first draft and with JR produced the final draft. All authors read and approved the final manuscript.
